# Probiotic Effects of a Novel Strain, *Acinetobacter* KU011TH, on the Growth Performance, Immune Responses, and Resistance against *Aeromonas hydrophila* of Bighead Catfish (*Clarias macrocephalus* Günther, 1864)

**DOI:** 10.3390/microorganisms7120613

**Published:** 2019-11-25

**Authors:** Anurak Bunnoy, Uthairat Na-Nakorn, Prapansak Srisapoome

**Affiliations:** 1 Laboratory of Aquatic Animal Health Management, Department of Aquaculture, Faculty of Fisheries, Kasetsart University, 50 Paholayothin Rd, Ladyao, Chatuchak, Bangkok 10900, Thailand; anurak.bunnoy@gmail.com; 2 Laboratory of Aquatic Animal Genetics, Department of Aquaculture, Faculty of Fisheries, Kasetsart University, 50 Paholayothin Rd, Ladyao, Chatuchak, Bangkok 10900, Thailand; ffisurn@ku.ac.th

**Keywords:** *Acinetobacter* KU011TH, *Clarias macrocephalus*, probiotics, growth performance, innate immune responses, skin physical barriers, immune-related gene, *Aeromonas hydrophila*

## Abstract

In the present study, the novel probiotic strain *Acinetobacter* KU011TH with an evident lack of pathogenicity in catfish was experimented. Three practical administration routes, namely, feed additive (FD), water-soluble additive (SOL), and a combination route (FD+SOL), were applied in two sizes of catfish. After 120 days of FD+SOL administration, catfish fingerlings (15 g) exhibited a significant improvement in all tested growth performance parameters. For 15- and 30-day applications at the juvenile stage (150 g), phagocytic activity, phagocytic index, lysozyme activity, respiratory burst activity, alternative complement pathway, and bactericidal activity were significantly increased. Furthermore, probiotic-administered bighead catfish exhibited an upregulated expression of several immune-related genes in tested organs. Significant colonization by *Acinetobacter* KU011TH in rearing water and on skin and gills was observed among experimental groups. Histological analysis clearly indicated enhanced physical characteristics of skin mucosal immunity in the treated groups. No histopathological changes in the gills, skin, intestine or liver were observed among the fish groups. Interestingly, after challenge with *Aeromonas hydrophila*, the survival rates of the treated groups were significantly higher than those of the controls. In conclusion, the novel probiont *Acinetobacter* KU011TH provides a potent strategy for improvement in growth and disease resistance, which is an important steppingstone for sustaining catfish aquaculture.

## 1. Introduction

In Thailand, catfish farming has been growing at an impressive rate in recent decades. Catfish production is the second largest fish aquaculture industry, after that of Nile tilapia (*Oreochromis niloticus*), with an average of approximately 17,900 million tons produced per year since 1990, reaching maximal production at 159,314 million tons in 2004 and subsequently decreasing to 107,730 metric tons in 2017 (Food and Agriculture Organization, FAO; http://www.fao.org/statistics/en/;1-12-2018) [1]. The bighead catfish (*Clarias macrocephalus* Günther, 1864)*,* known as broadhead catfish or “pla-duk-uey” in Thai, is an economically important cultured species in Southeast Asia, especially Thailand. This species provides high-quality meat with unique flavors. Consequently, female *C. macrocephalus* fish have been used for producing hybrid catfish (*C. macrocephalus* x *Clarias gariepinus*), known as “pla-duk-big-uey” [2]. Specifically, hybrid catfish have demonstrated many commercially desirable characteristics, such as rapid growth; increased feed conversion, resistance to infectious diseases, and overall survival, as well as their tolerance to a wide range of environmental conditions. As a consequence, these fish have attained a remarkable market value in production and export among the fish and fishery products of Thailand [2,3]. Unfortunately, a main problem of the hybrid parent species, bighead catfish, is high susceptibility to infectious diseases and sensitivity to various environmental conditions [4]. A dramatic decrease in the population of these fish has become a serious issue in hybrid catfish farming and production. The recent extensive demands and increased output of catfish are likely to require an intensive aquaculture system. Intensively farmed catfish are occasionally subjected to stressful conditions, leading to increased susceptibility to pathogens, especially *Aeromonas hydrophila* and *Flavobacterium columnare,* which also affect a wide variety of freshwater fish species [3,5]. Accordingly, disease outbreaks continue to be a major problem for the sustainable development of aquaculture industries. Traditionally, antibiotics and chemicals have been the most common and extensively used therapies to treat and control diseases of infected aquatic animals [6]. Nevertheless, the adverse effects of these substances have been a concern for many years because the excessive usage of antibiotics and chemicals could lead to drug-resistant pathogens, food and environmental contamination and the continuously rising cost of disease treatment [2,3]. Consequently, interest in alternative disease control strategies, including nonspecific immunostimulants, vaccines, nutritional supplements, phytochemical products, and probiotics, has been increasing in the global aquaculture industry during the last decade [7].

Probiotics, defined by Fuller [8] as “a live microbial feed supplement which beneficially affects the host animal by improving its microbial balance,” beneficially affect the host by enhancing growth performance, stimulating the immune system and improving water quality in aquatic environments [9]. Various microorganism species have been reported and introduced as probiotics in the aquaculture industry, including Gram-negative bacteria such as those of the genera *Aeromonas, Enterobacter, Pseudoalteromonas, Pseudomonas, Rhodopseudomonas,* and *Vibrio,* and Gram-positive bacteria such as those of the genera *Bacillus, Enterococcus, Lactobacillus, Lactococcus, Microbacterium, Micrococcus, Streptococcus,* and *Streptomyces* [10,11]. However, the isolation and efficacy of probiotic microorganisms that originated from bighead catfish (*C. macrocephalus* Günther, 1864) have not been reported thus far.

*Acinetobacter* KU011TH is a microorganism that was successfully isolated from the skin mucus of bighead catfish (*C. macrocephalus* Günther, 1864), and then, genome sequence analysis classified it as a novel bacterial species of the genus *Acinetobacter* [12]. *Acinetobacter* KU011TH is a Gram-negative coccobacillus that is immotile, aerobic, catalase positive, oxidase negative, and nonhemolytic and does not form spores. This species has the ability to grow under a wide range of environmental conditions, at temperatures ranging from 4–41 °C, in pH values ranging from 3.0–10.0, and in the presence of 0–10 (w/v) NaCl. Optimal growth of the strain was observed at 30–32 °C, in pH 7.0–8.0 and in the presence of 1.0% (w/v) NaCl. Interestingly, *Acinetobacter* KU011TH exhibits a wide range of efficacy in antagonistic activity against various freshwater fish pathogens in agar dot-spot and coculture assays, including against *A. hydrophila, F. columnare, Flectobacillus roseus, Streptococcus agalactiae, Staphylococcus warneri* and *Edwardsiella tarda*. Additionally, the strain could also grow in saline conditions, with potent activity against marine fish pathogens, including *Vibrio alginolyticus, Vibrio harveyi, Vibrio parahaemolyticus* AHPND, and *Vibrio vulnificus* [12].

Therefore, the aim of this study was to investigate the efficacy of *Acinetobacter* KU011TH on growth performance, innate immune responses, skin mucosal characteristics, immune-related gene expression and disease resistance in bighead catfish. The data obtained from this research could be further applied by replacing antibiotics used in the catfish aquaculture industry.

## 2. Materials and Methods

### 2.1. Cultivation and Preparation of the Probiotic Bacterium

The *Acinetobacter* KU011TH bacterium was obtained from the Laboratory of Aquatic Animal Health Management, Department of Aquaculture, Faculty of Fisheries, Kasetsart University, Thailand. This bacterium was entirely described in terms of both genomic and phenotypic information in [12]. The bacterium was routinely grown on plate count agar (PCA, HiMedia Laboratories) at pH 7.3 and incubated at 32 °C for 18–24 h. After incubation, a purified single colony was selected, cultured in a sterilized 50-mL tube in nutrient broth (NB) medium containing 0.5% tryptone, 0.3% yeast extract and 1.0% dextrose/glucose at pH 7.3 and incubated at 32 °C for 18–24 h. Bacterial pellets were obtained by centrifugation at 2500 rpm for 10 min. Bacterial cells were then washed twice in 0.85% NaCl. The bacterial cell density was measured using a spectrophotometer (Thermo Fisher Scientific, MA, USA), monitoring the optical density at 600 nm. Microscopic examinations of colony and cell morphologies were performed to confirm the purity of the strains before all experiments.

### 2.2. Analyses of Probiotic Shelf Life and Its Efficacy Against Fish Pathogens

To determine how long the probiotic *Acinetobacter* KU011TH maintained viable efficacy when preserved at 4 °C for further practical application, a study of bacterial shelf life and its efficacy against the pathogen *A. hydrophila* was carried out prior to in vitro trials. Bacterial cells were prepared as described above. Briefly, bacterial cells were suspended in 0.85% (w/v) NaCl at approximately 1 × 10^7^ colony-forming units (CFU)/mL, and then, one milliliter of bacterial suspension was transferred to thirty different 1.5-mL tubes. All bacteria-containing tubes were then refrigerated at 4 °C for ten weeks of trials. The shelf life and viable efficacies of the probiotics were measured weekly using bacterial colony counting on PCA plates at 24 h [12]. Additionally, the efficacy of the probiotic against the pathogen *A. hydrophila* was assessed using an agar dot-spot assay. The diameters of clear zones around the probiotic bacteria were measured in centimeters [12]. All tests were performed weekly, with three replications. The viable cell counts and clear-zone diameters were compared to those at day 0 (control) of the experiment.

### 2.3. Safety and Pathogenicity Assessments of Acinetobacter KU011TH on C. macrocephalus

Whether the novel bacterial strain was nonpathogenic to the host bighead catfish was assessed through median lethal dose (LD_50_) and median lethal concentration (LC_50_) toxicity tests by injection and immersion strategies, respectively, according to [13]. A pathogenicity test was conducted on healthy catfish weighing 100 ± 5 g to determine the LD_50_ and LC_50_ of *Acinetobacter* KU011TH. After acclimatization, fish were randomly divided into nine different tanks (10 fish/tank) with three replications for LD_50_ or LC_50_ tests. *Acinetobacter* KU011TH suspensions were prepared as described above. For the LD_50_ toxicity assay, bacterial suspensions were intraperitoneally injected (0.1 mL of 1 × 10^2^, 10^4^, 10^6^, 10^8^, 10^10^, 10^12^, 10^14^ or 10^16^ CFU/mL *Acinetobacter* KU011TH), and a control group was injected with 0.1 mL of 0.85% NaCl. For the LC_50_ toxicity assay, fish were immersed with bacterial suspensions at 1 × 10^2^, 10^4^, 10^6^, 10^8^, 10^10^, 10^12^, 10^14^ and 10^16^ CFU/mL *Acinetobacter* KU011TH. After exposure, cumulative mortality and clinical signs were monitored at 12-h intervals for 15 days. Histopathological examination of treated and control fish was also performed on the target organs, including the liver, spleen, head kidney and gastrointestinal (GI) tract, to confirm safety at the cellular level. Feeding was optimized during the experiment.

### 2.4. Preparation of the Acinetobacter KU011TH-Supplemented Diets and Water-Soluble Bacteria

A bacterial suspension was prepared for two practical uses, namely for diet supplementation and for water-soluble administration, to determine the efficacy of *Acinetobacter* KU011TH in diet supplementation, water-soluble administration, and the combination of the two administration routes in bighead catfish culture conditions.

For the diet supplementation of bacteria, three different concentrations (1 × 10^5^, 1 × 10^7^ and 1 × 10^9^ CFU/mL) in a total volume of 200 mL were also prepared as described above. Bacterial suspensions were separately mixed with 1.0 kg of commercial diet (Betagro Ltd., Thailand) and slowly mixed in a mixer, resulting in 1 × 10^5^, 1 × 10^7^, and 1 × 10^9^ CFU/kg diet for the respective diet supplementation tests. The mixed diets were then coated with 2.0% (v/w) sterile squid oil. Then, the mixed diets were allowed to air dry at room temperature for 2 h to remove the excess moisture from the diets and stored at 4 °C for all experimental use. The proximate compositions of the diets were analyzed by Betagro Ltd., Thailand. The major compositions are 40% crude protein, 12% moisture, 4% crude fat, 4% crude fiber, 1.0% amino acids, 1.5% minerals and 1.0% vitamins. No significant differences of each nutritional profile among tested diets were observed.

The preparation of a bacterial suspension for diet supplementation and for water-soluble administration was routinely performed at three-day intervals. To ensure appropriate probiotic levels in the diet, the retention and variability of the supplemented bacteria in the diet were monitored on day 3 after feed mixing by counting colonies on PCA plates as described above.

For water-soluble administration, three different concentrations (1 × 10^6^, 1 × 10^7^ and 1 × 10^8^ CFU/mL) of the probiotic *Acinetobacter* KU011TH in a total volume of 200 mL were prepared as described above. Bacterial suspensions were then directly administered into the rearing water in fish tanks containing 200 L of aerated water, resulting in final concentrations of 1 × 10^3^, 1 × 10^4^ and 1 × 10^5^ CFU/mL for the respective water-soluble administration tests.

### 2.5. Experimental Fish and Conditions

Healthy bighead catfish (*C. macrocephalus* Günther, 1864) fingerlings weighing 15 g and juveniles weighing approximately 150 g were obtained from the Department of Aquaculture, Faculty of Fisheries, Kasetsart University, Thailand. The fingerling and adult catfish were assessed with the same procedures but based on different trial parameters by monitoring growth (120-day trial) and immune responses (30-day trial), respectively. Catfish were acclimatized in a quarantine tank with aerated freshwater at a temperature of 28 ± 2 °C and pH of 7 ± 1 for 15 days before the experiment trial, during which their health conditions were monitored. The photoperiod was 12 h light:12 h dark. The fish were fed a commercial diet without probiotics (Betagro Ltd., Thailand) twice a day at a rate of 2% of their body weight. Uneaten feeds and unwanted sediment were regularly removed.

After the acclimatization period, fish were removed to avoid microbial contamination of the fish and water before initiation of the probiotic experimental trials; the catfish were immersed in a 100-ppm formalin solution for one minute and then randomly separated into 250-L tanks containing 200 L of water. The temperature, dissolved oxygen content and pH of the rearing water were measured weekly using an EcoSense EC300M, EcoSense DO200A and EcoSense pH100M (YSI Environmental, USA), respectively. Chemical parameters, such as ammonia, nitrite and nitrate levels, were also measured using commercial kits (Advance Pharma, Bangkok, Thailand) according to the manufacturer’s instructions.

The experimental procedures performed with aquatic animals were carried out in accordance with the Ethical Principles and Guidelines for the Use of Animals National Research Council of Thailand for the care and use of animals for scientific purposes. The protocol was approved by the Animal Ethics Committee, Kasetsart University, Thailand (ethics ID: ACKU61-FIS-004).

### 2.6. Effects of Acinetobacter KU011TH Application on Growth Performance after 120 Days of Trial and on Innate Immune Responses, Immune-Related Gene Expression, and Skin Physical Characteristics of C. macrocephalus after 30 Days of Trial

#### 2.6.1. Experimental Designs

In this experiment, for ideal results, fish were separated based on the two main purposes, with different trial parameters, but with the same experimental procedures: 1) fingerling fish weighing 15 g were used to determine the effect of the *Acinetobacter* KU011TH bacterium on growth for a 120-day trial, and 2) juvenile fish weighing 150 g were used to determine the effect of the bacterium on fish immune responses for a 30-day trial.

A completely randomized design (CRD) of all trials with three replicates was performed. Acclimatized healthy fish were assigned to ten experimental groups. Fish in group 1 (control) were treated with only commercial (control) diet without administration with probiotic bacteria in the diet or rearing water. Fish in groups 2–4 (FD5, FD7 and FD9) were subjected to only diet supplementation with *Acinetobacter* KU011TH at 1 × 10^3^, 1 × 10^5^ and 1 × 10^7^ CFU/kg diet, respectively, without administration of probiotic bacteria in the rearing water. Fish in groups 5–7 (SOL3, SOL4 and SOL5) were treated with commercial (control) diet and administration only the water-soluble form of the probiotic bacterial suspension via the rearing water at 1 × 10^3^, 1 × 10^4^ and 1 × 10^5^ CFU/mL rearing water, respectively. Fish in groups 8–10 (FD5+SOL3, FD7+SOL3 and FD9+SOL3) were combinatorially treated with *Acinetobacter* KU011TH via diet supplementation with 1 × 10^3^, 1 × 10^5^ and 1 × 10^7^ CFU/kg diet, respectively, and with the water-soluble form of the probiotic bacteria via the rearing water at 1 × 10^3^ CFU/mL water. All experimental groups contained 60 fish, with three replicates (20 fish/replicate), and were reared in 200-L aerated-water tanks. Approximately 30% of the water in all the tanks was exchanged weekly. Water-soluble bacterial suspensions with different concentrations were directly added to 200 L of rearing water in the tanks of groups 4–10 (SOL3, SOL4, SOL5, FD5+SOL3, FD7+SOL3 and FD9+SOL3) in the respective trials, as described above. For the control group, an equal volume of a 0.85% NaCl solution was added instead of water-soluble bacterial suspensions. Soluble bacterial suspensions were added at three-day intervals until the end of the experimental period. Additionally, fish were manually fed with the control diet or different supplemented diets twice a day, with a feeding rate of 2.0% body weight for the entire experimental period. The daily feed rate was adjusted every seven days by random weighing.

#### 2.6.2. Collection of Peripheral Blood Lymphocytes (PBLs) and Fish Tissues

The effects of *Acinetobacter* KU011TH on innate humoral and cellular immune responses were assessed over 30 days, and sampling was performed three times: before starting the experiment (day 0), in the middle of the experiment (day 15) and at the end of experimental trials (day 30). Nine fish per treatment (three fish per tank) at each sampling time were weighed and collected to determine the innate immune parameters and immune-related gene expression levels and to quantify colonization by *Acinetobacter* KU011TH of the rearing water, gills, skin and intestine at 0, 15, and 30 days. Moreover, measurement of the skin mucus cells of catfish and histopathology of the gill, skin, liver, and intestine tissues was conducted once at 30 days.

One milliliter of blood was collected from the caudal vein of each fish with a 1.0-mL syringe. Five hundred microliters of blood from each fish was transferred to 1.5-mL microcentrifuge tubes without anticoagulant solution and containing an anticoagulant solution (heparin) to collect the serum and PBLs, respectively. The non-anticoagulant tubes were kept at room temperature for 2 h and then centrifuged at 3000 × g for 10 min. The obtained serum was stored at −80 °C for further use. For tubes containing five hundred microliters of anticoagulant, PBLs were separated from other blood components using the Ficoll method [14]. The purified PBLs were resuspended in phosphate-buffered saline (PBS; pH 7.4) and transferred to a 1.5-mL microcentrifuge tube at 4 °C for further experiments.

Approximately 25 mg of the gill, liver and head kidney tissues of each fish were aseptically collected and immediately preserved in 1.0 mL of NucleoZOL^TM^ reagent (Clontech Laboratories, CA, USA). Following the recommended protocol, mRNAs of these tissues were extracted and reverse transcribed to synthesize first strand cDNAs to determine immune-related gene expression. For DNA extraction and quantification of *Acinetobacter* KU011TH colonization in target organs and rearing water, DNA isolation was conducted by collecting approximately 25 mg of the gill, skin and head kidney tissues of each fish and 1 mL of rearing water from each tank. DNA extraction from tissues and water was performed using DNAzol reagent (Thermo Fisher, CA, USA) and the GeneJET Genomic DNA Purification Kit (Thermo Fisher, CA, USA) according to the manufacturer’s instructions. Additionally, small pieces of gills, skin, intestine and liver were also collected, fixed in a 10% (v/v) buffered formalin solution and processed for histological assessment [15].

#### 2.6.3. Effects of *Acinetobacter* KU011TH on the Growth Performance of Bighead Catfish at 120 Days

The effects of the *Acinetobacter* KU011TH bacterium on growth performance were assessed using fingerling fish weighing 15.0 ± 2.0 g with a body length of 7.0 ± 3.0 cm at the beginning of a 120-day trial. The growth performances of all treatment groups were based on their body weight and length data. Thirty fish from each treatment group (10 fish per tank) were randomly monitored for growth performance parameters by weighing body weight and measuring the total length monthly. Growth performances were reported as 1) absolute growth rate (AGR), including total weight gain (WG) or length gain (TLG), and average daily growth rate (ADG), 2) relative growth rate (RGR), and 3) specific growth rate (SGR). Moreover, the feed conversion ratio (FCR) was also measured in all experimental trials. All calculations of growth were performed using the following methods as described by [16]:

WG or TLG (g or cm /120 days) = *W_t_ -W_i_* (1)

ADG (g or cm / day) = (*W_t_ -W_i_*) */ t* (2)

RGR (% in 120 days) = (*W_t_ -W_i_*) */ W_i_* × *100* (3)

SGR (% / day) = *log* (*W_t_*) *– log (W_i_*) */ t* × *100* (4)

FCR = amount of total feed given / WG(5)

where *W_t_* is the final weight/length, *W_i_* is the initial weight/length, and *t* is the duration of the trial (120 days).

The amount of diet consumed was monitored by drying and weighing daily excess feed. Daily feeding rations were adjusted every 15 days by randomly batch weighing experimental fish.

#### 2.6.4. Effects of *Acinetobacter* KU011TH on the Innate Immune Response of Bighead Catfish

##### Lysozyme Activity Analysis

The lysozyme activity of the serum was determined according to the method of [17] based on lysis of *Micrococcus lysodeikticus* (Sigma, MO, USA). The units of lysozyme activity were calculated as units/mL enzyme = [(*Δ A_540_* sample - *Δ A_540_* blank) × dilution factor]/ (0.001) (0.1)

##### Respiratory Burst Reaction Analysis

The respiratory burst activity of phagocytes was measured using nitroblue tetrazolium (NBT) dye reduction to quantitate superoxide anion, which is one of the first reactive oxygen intermediates (ROIs) produced by phagocytes. The NBT reaction was conducted following methods described by [18]. Color development of the NBT dye reactions was monitored at 650 nm.

##### Hemolytic Activity by the Alternative Complement Pathway (ACH_50_) Assay

Hemolytic activity was determined using the alternative complement pathway (ACH_50_) assay following the method described by [19], using sheep red blood cells (ShRBCs) as target cells for hemolysis. Serially diluted plasma of each fish sample was incubated with ShRBCs (1 × 10^8^ cells/mL) in round-bottomed 96-well plates at 32 °C for 3 h. Then, 50 μL of the supernatants was transferred to new flat-bottomed 96-well plates after centrifugation at 2500 rpm for 5 min, and the absorbance of the supernatant was measured at 540 nm. Incubation of ShRBCs with distilled water was performed as a positive control (100% lysis), and ShRBCs without serum were used as blanks or negative controls (0% lysis). The reciprocal of the serum dilution that caused 50% lysis of ShRBCs was designated ACH_50_. The results are presented as ACH_50_ units/mL fish serum based on the value of *K,* which was obtained from *Y*/(1-*Y*), where the value of *Y* was defined as the ratio of adjusted serum hemolysis to complete hemolysis against the reciprocal of the serum dilution as follows:

ACH_50_ units/mL = 1/K x reciprocal of the serum dilution × 0.2 (6)

where K is the amount of serum (mL) that caused 50% lysis, and 0.2 is the correction factor of the original method.

##### Bactericidal Activity Analysis

A septicemia-causing pathogenic bacterium of catfish, *A. hydrophila*, was used to determine the bactericidal activity of the serum samples. Purified bacteria were grown on tryptic soy agar (TSA) plates at 32 °C for 18 h. A single colony from the incubated plate was then subcultured in 50 mL of tryptic soy broth (TSB). Cell pellets were obtained by centrifugation at 2500 rpm for 10 min. A bacterial suspension with a concentration of 1 × 10^3^ CFU/mL was prepared and adjusted by measuring the absorbance at 600 nm. The bactericidal activity of the experimental fish serum was assessed by determining the survival of bacteria after incubation with serum [20]. Briefly, one hundred microliters of fish serum were cultured with 100 μL of a bacterial suspension of *A. hydrophila* in flat-bottomed 96-well plates and incubated at 32 °C for 1 h prior to the enumeration of *A. hydrophila* survival by plate counting on TSA plates after 24 h. The bactericidal activity of fish serum was expressed as the survival of *A. hydrophila* after exposure to fish serum relative to that of an experimental control group. Samples without bacteria and samples without serum were used as blanks (negative control) and positive controls (100% survival or 0% bactericidal activity), respectively.

##### Phagocytic Activity Analysis

The phagocytic activity of blood leukocytes was determined using a previously described method [21,22] with some modifications. Briefly, 200 μL of PBLs at approximately 1 × 10^8^ cells/mL for each sample were loaded onto 22 × 22 mm cover glass slides with three replicates and allowed to adhere on the glass surface for 2 h. Cells were washed with RPMI twice to eliminate unadhered cells. An approximately 1 × 10^7^ particle/mL latex bead (Sigma-Aldrich, USA) suspension was then loaded onto the adhered cells on the cover glass slide. Excessive bead particles were washed with RPMI twice at 2 h. Cells were stained with a Dip-Quick staining kit (VR Bioscience, Thailand) and analyzed under a microscope, counting 300 cells in total. Phagocytic activity (PA) and phagocytic index (PI) were calculated using the following formula:

PA = (number of phagocytic cells/number of total cells count) × 100.(7)

PI = (number of total bead particles/number of total phagocytic cells) × 100.(8)

#### 2.6.5. Effects of *Acinetobacter* KU011TH on Immune-Related Gene Expression of Bighead Catfish

Total RNA from three organs, including the gills, liver and skin, was extracted by homogenization in a cell disrupter (MP FastPrep 24, CA, USA) using 1.0 mL of NucleoZOL^TM^ reagent (Clontech Laboratories, CA, USA) according to the manufacturer’s protocol. Total RNA concentration was determined using a NanoDrop^TM^ spectrophotometer (Thermo Fisher Scientific, MA, USA). One microliter of 1000 ng/μL total RNA was then used as a template to synthesize first-strand cDNA using the ReverTra Ace qPCR RT Master Mix with gDNA Remover Kit (TOYOBO, Japan) according to the manufacturer’s protocol. The product of the first-strand cDNA synthesis was stored at −80 °C for further experiments.

Immune-related genes of the innate immune response, namely, alpha-2-macroglobulin (*A2M*), CC chemokine (*CC*), C3 complement (*C3*), lysozyme C (*LyzC*), myeloperoxidase (*MPO*), nuclear factor kappa B (*NF-kB*) and bactericidal/permeability-increasing protein (*BPIP*), were used to assess immune-related gene expression [3,22,23,24,25,26,27]. The primers used in the study were designed and obtained from the transcriptome project of liver tissues of the bighead catfish, *C. macrocephalus* (accession number SRS1284019). The primers used in the present study are listed in Table 1.

The quantification of immune-related gene expression was performed using quantitative reverse transcriptase PCR (qRT-PCR) assays. A qRT-PCR assay was performed with Brilliant III Ultra-Fast SYBR Green (Agilent, CA, USA) in an Mx3005P QPCR Systems instrument (Agilent, CA, USA). The qPCRs were optimized in 20-μL reactions containing 10 μL of 2 × SYBR Green QPCR Master Mix, 2 μL of 0.5 mM forward and reverse primers, and 1 μL of cDNA template, adjusting the reaction mixture with distilled water to a final volume of 20 μL. The qPCR cycling conditions included initial conditions of one cycle of 95 °C for 5 min; 40 cycles of 95 °C for 30 s, 55 °C for 30 s, and 72 °C for 90 s; and a final extension at 72 °C for 10 min. Triplicate qRT-PCRs were carried out for each sample. The *β-actin* gene was used as the housekeeping gene to standardize the results by eliminating variation in mRNA and cDNA quantity and quality. The relative expression of immune-related genes in catfish tissues at different time points was calculated using 2^-ΔΔCT^ analysis according to the protocol of [28]. The level of statistical significance between the control and treatment groups was indicated as * (*p* < 0.05) and ** (*p* < 0.01) using Student’s t-test.

#### 2.6.6. Quantification of *Acinetobacter* KU011TH in the Rearing Water, Skin, Gills and Intestine of Bighead Catfish

To enumerate the *Acinetobacter* KU011TH in the experimental systems, genomic DNA of strain KU011TH in target organs and rearing water was obtained as described above. Quantification of bacterial numbers was performed using a qPCR assay. Specific primers for the DNA gyrase subunit B (*gyrB*) gene of *Acinetobacter* KU011TH were obtained from its whole-genome sequence (accession number MG950236) and are listed in Table 1.

Samples were subjected to a qPCR assay using Brilliant III Ultra-Fast SYBR Green (Agilent, CA, USA) in an Mx3005P QPCR Systems instrument (Agilent, CA, USA). The qPCRs were previously described. The specificity and efficiency of the primers were evaluated by conventional PCR and real-time PCR, respectively. Using the above optimized real-time PCR amplification conditions, a standard curve for quantification of *Acinetobacter* KU011TH was prepared using 10-fold DNA serial dilutions from 1 × 10^2^ to 1 × 10^10^ copies/μL. The threshold cycle (*C_T_*) was measured for each qPCR. The obtained *C_T_* values of the samples were then plotted against the number of microorganisms. The resulting values (DNA copy numbers) were calculated from the standard equation for a target strain. All tests and qPCRs were run in triplicate.

#### 2.6.7. Effects of *Acinetobacter* KU011TH on the Histopathology of the Gills, Skin, Intestine and Liver of Bighead Catfish

After euthanasia, three fish per treatment were dissected, and their gills, skin, and middle intestine were collected and fixed in a 10% buffered formalin solution for 24 h. After fixation, the samples were cross-sectioned, dehydrated and embedded in paraffin blocks according to standard histological procedures [15,29]. Three-micron thick sections were stained for histological purposes with hematoxylin and eosin (H&E). The histopathology of the tissues was examined with a light microscope with DP2-BSW live imaging software (Olympus, MA, USA).

#### 2.6.8. Effects of *Acinetobacter* KU011TH on the Skin Epithelium and Skin Mucus Cells of Bighead Catfish

The skin epithelium and skin mucus cells were analyzed using the obtained skin histological sections of 3 different fish in each treatment at day 30 after application. The skin histological images of each sample were subjected to analysis of skin mucosal tissues, which are described in [30]. The skin thickness and areas of interest were randomly outlined on skin epithelial tissues for up to three different areas per section using Olympus live imaging software (DP2-BSW, Olympus, MA, USA). Skin epithelial thickness (μm), number of mucous cells/μm^2^ epithelial area, mucus cell areas/μm^2^ epithelial area, mucus cell density in the epithelium (%), and the ratio of epithelial cell areas to mucus cell areas (E:M) were obtained using the Materials Image Processing and Automated Reconstruction (MIPAR™) software [31].

#### 2.6.9. Effects of *Acinetobacter* KU011TH on Disease Resistance of Bighead Catfish against *A. hydrophila*

At the end of the experimental trial period (day 30), thirty fish from each treatment (ten fish per tank) were randomly separated for a challenge test with *A. hydrophila.* A virulent pathogenic bacterium, *A. hydrophila* AQH0018, was prepared as described above. *A. hydrophila* was identified based on bacterial morphology and biochemical properties. The preliminary pathogenicity of virulent *A. hydrophila* toward *C. macrocephalus* was assessed to validate the optimum dose for the experimental challenge trials. The seven-day median lethal dose (LD_50_) was assessed by intraperitoneal injection with 0.1 mL of graded doses of *A. hydrophila* at 1 × 10^5^, 10^6^, 10^7^, 10^8^ and 10^9^ CFU/mL into 10 fish. The LD_50_ of virulent *A. hydrophila* toward *C. macrocephalus* was approximately 1 × 10^6^ CFU/mL. For the infection experiment, the obtained optimal dose was used for the challenge test in the trial. All fish were intraperitonially injected with 0.1 mL of virulent *A. hydrophila* at 1 × 10^6^ CFU/mL. The mortality was recorded every 6 h for up to 15 days post challenge. Re-isolation of *A. hydrophila* from moribund fish was performed to verify the cause of the death in fish using a standard diagnostic protocol [32].

#### 2.6.10. Statistical and Data Analysis

The obtained growth performance, innate immune response, bacterial colonization, and skin mucosal immunity data were obtained for three replicates, and the results are expressed as the mean ± standard error of the mean (SEM). Data were statistically analyzed by one-way analysis of variance (ANOVA) and Duncan’s new multiple range test (DMRT) were used to determine differences among groups at days 15 and 30 of the trials, respectively. All statistical analyses were performed using Statistical Package for Social Science (SPSS for Mac version 24.0, Chicago, IL, USA). The level of statistical significance between the control and treatment groups at different times checked was indicated as * (*p* < 0.05) and ** (*p* < 0.01) using Student’s t-test.

Survival analysis of bighead catfish in each group was calculated using the Kaplan-Meier method [33]. Cumulative survival plots were generated using SPSS for Mac (version 24.0, Chicago, IL, USA). The level of statistical significance between the control and treatment groups was indicated as * (*p* < 0.05) and ** (*p* < 0.01) using Student’s *t*-test.

## 3. Results

### 3.1. Analysis of the Bacterial Shelf Life of the Probiotic and its Efficacy Against Pathogens

The shelf life and efficacy of *Acinetobacter* KU011TH against *A. hydrophila* after preservation at 4 °C for ten weeks are presented in Table 2. The total viable bacterial counts were not significantly decreased from the first week until the fourth week, with an approximate decrease of 3.15% to 4.87% (*p* > 0.05). After four weeks of preservation, the viable bacterial counts decreased slightly by 21.17%, 34.22%, 45.69%, 49.29%, 46.28% and 52.63% from weeks 5 to 10, respectively, compared to those of the control (week 0). However, there were no significant differences in the bacterial numbers from weeks 7 to 10 (*p* > 0.05). Additionally, the efficacy of *Acinetobacter* KU011TH against the pathogen *A. hydrophila* each week was not significantly different in terms of inhibitory clearance zone diameters compared to that of the control (week 0) (*p* > 0.05).

### 3.2. Safety and Pathogenicity Assessments of Acinetobacter KU011TH on C. macrocephalus

Upon isolation and characterization of the novel bacterial species *Acinetobacter* KU011TH from the skin mucus of bighead catfish (*C. macrocephalus*), the pathogenicity of the host was unsuccessfully assessed through LD_50_ and LC_50_ values in this study. Mortality, clinical and pathological features of swimming behavior and skin and gill appearances were not observed over the entire period in either control or treated fish during the trials. The values of LD_50_ and LC_50_ could not be obtained for the *Acinetobacter* KU011TH bacterium, even when the bacteria were applied at 1 × 10^16^ CFU/mL. Indeed, no histopathological changes were observed in any of the groups of fish (data not shown). Notably, the novel strain was proven to be a nonpathogenic bacterium toward the host, which warrants further study.

### 3.3. Effect of a Probiotic Bacterium on Growth Performance in a 120-Day Trial

The effects of *Acinetobacter* KU011TH on the growth performance of *C. macrocephalus* are presented in Table 3. After a 120-day trial, fish treated with only a combination of an *Acinetobacter* KU011TH*-*supplemented diet at 1 × 10^9^ CFU/kg diet and water-soluble bacteria at 1 × 10^3^ CFU/mL (FD9+SOL3) showed a significant increase in WG, ADG, RGR, SGR and FCR for body weight compared with those of control groups (*p < 0*.05). However, there were no significant differences in growth parameters among the control, FD5, FD7, FD9, SOL3, SOL4, SOL5, FD5+SOL3 and FD7+SOL3 groups after the 120-day trial. Additionally, no significant difference was observed in the TLG, ADG, RGR, and SGR for body length in any of the treatments. The fish survival rate after the 120-day trial period was not significantly different among the groups, with averages of 80.24 ± 5.67% to 85.97 ± 6.77% (data not shown).

### 3.4. Effect of a Probiotic Bacterium on Innate Immune Responses, Immune-Related Gene Expression, and Skin Characteristics of C. macrocephalus

#### 3.4.1. Humoral and Cellular Immune Responses of Innate Immunity

**Figure 1:**
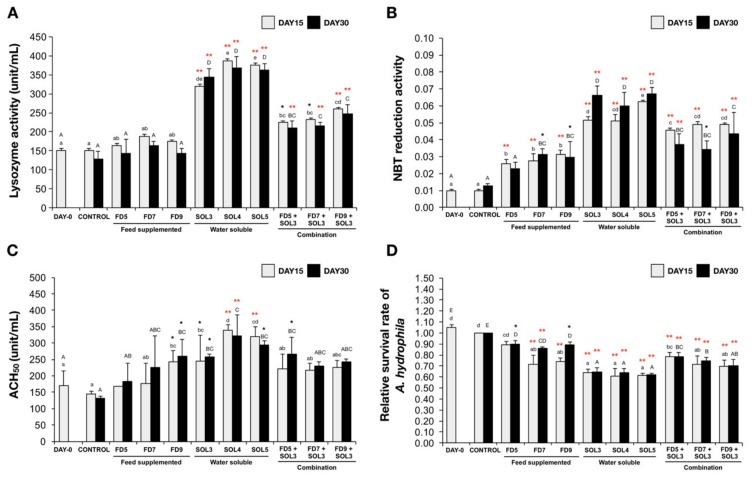
Effect of *Acinetobacter* KU011TH administration on the humoral and cellular innate immune responses of bighead catfish. Lysozyme activity (**A**), respiratory burst activity (**B**), ACH_50_ (**C**) and bactericidal activity (**D**) values are presented as the mean ± SEM (*n* = 9). Lowercase and uppercase letters indicate differences among treatment groups at 15 and 30 days of the trial, respectively. The levels of statistical significance between the control and treatment groups are indicated by * (*p* < 0.05) or ** (*p* < 0.01).

**Figure 2:**
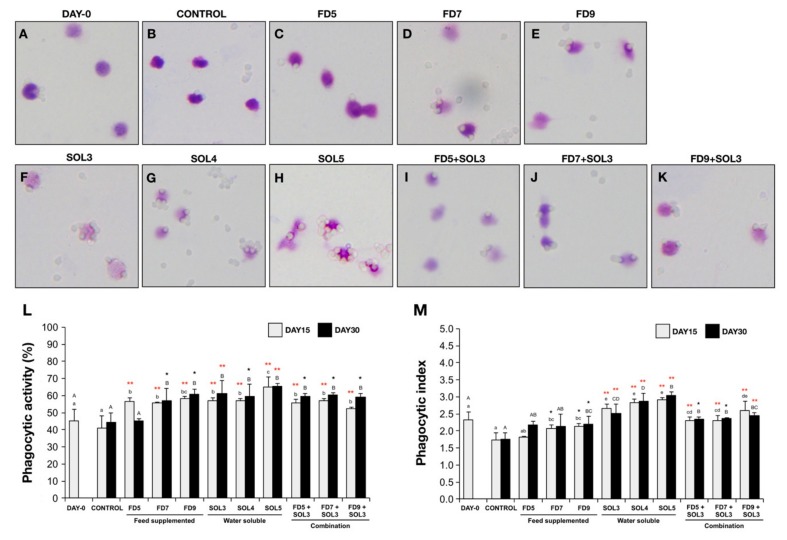
Microscopic images of phagocytosis assays show the adhered and engulfed bead particles of phagocytic cells of non- *Acinetobacter* KU011TH-administered fish (**A**–**B**) and fish administered *Acinetobacter* KU011TH through feed (**C**–**E**), water (**F**–**H**) or a combination (**I**–**K**). Phagocytic activity (**L**) and phagocytic index (**M**) values of phagocytes of bighead catfish (*C. macrocephalus*) are presented as the mean ± SEM (*n* = 9). Lowercase and uppercase letters indicate significant differences among treatment groups at 15 and 30 days of the trial, respectively. The levels of statistical significance between the control and treatment groups are indicated by * (*p* < 0.05) or ** (*p* < 0.01).

The effects of *Acinetobacter* KU011TH on innate immune responses, including the PA, lysozyme activity, respiratory burst reaction, ACH_50_ and bactericidal activity, of *C. macrocephalus* are presented in Figure 1 and Figure 2. The lysozyme activity of fish administered water-soluble bacteria and the combination with *Acinetobacter* KU011TH*-*supplemented diets in the SOL3, SOL4, SOL5, FD5+SOL3, FD7+SOL3 and FD9+SOL3 groups was significantly higher than that of the fish in the control group (*p* < 0.05 or *p* < 0.01), but the lysozyme activity of fish in the FD5, FD7 and FD9 groups was not significantly different from that of the fish in control group (*p* > 0.05) after 15 and 30 days of the trial. 

Notably, the highest lysozyme activity was also observed in the groups of fish treated with water-soluble bacteria (SOL), namely, SOL3, SOL4 and SOL5, increasing the lysozyme activity by approximately 2.4-, 2.5-, and 2.6-fold, respectively, compared to that of the fish in the control group at both 15 and 30 days of the trial (Figure 1A).

The respiratory burst (NBT) reaction of all the treatment groups had a statistically significant increase when compared to that of the control groups after 15 and 30 days of the trial (*p* < 0.05 or *p* < 0.01), but no significant difference in the respiratory burst reaction was observed in only the FD5 group after 30 days of the trial compared to that of the control group (*p* > 0.05). The groups of fish treated with water-soluble bacteria (SOL), namely, SOL3, SOL4 and SOL5, exhibited the highest NBT reductions of 3.1-, 2.8, and 3.2-fold, respectively, compared to those of the control group at both 15 and 30 days of the trial (Figure 1B).

The ACH_50_ levels of fish in the FD9, SOL3, SOL4 and SOL5 groups were significantly higher than those of fish in the control groups after 15 days of the trial (*p* < 0.05 or *p* < 0.01). Moreover, the ACH_50_ levels of fish in the FD9, SOL3, SOL4, SOL5 and FD5+SOL3 groups were also significantly higher than those of fish in the control groups after 30 days of the trial (*p* < 0.05 or *p* < 0.01). There was no significant difference in two feed supplementation groups (FD5 and FD7) compared to the control groups (*p* > 0.05) (Figure 1C).

The serum bactericidal activities of all experimental groups, with the exception of the FD5 group and only day 15, produced a significant decrease in the relative survival rate of *A. hydrophila* in the bactericidal assay compared to that of the control group after 15 and 30 days of the trial (*p* < 0.05 or *p* < 0.01). Notably, the greatest decrease in *A. hydrophila* abundance was observed in the groups of fish treated with water-soluble bacteria (SOL), namely, SOL3, SOL4 and SOL5, compared to the control, diet supplementation and combination groups (Figure 1D). 

The PAs of all experimental treatment groups are illustrated in Figure 2A–K. The PAs of all treatment groups (with the exception of FD5 at day 30) were significantly higher than those of the control groups after 15 and 30 days of the trial (*p* < 0.05 or *p* < 0.01) (Figure 2L). Additionally, the PI was significantly increased in the FD7, FD9, SOL3, SOL4, SOL5, FD5+SOL3, FD7+SOL3 and FD9+SOL3 groups after 15 days of the trial and remained increased in the FD9, SOL3, SOL4, SOL5, FD5+SOL3, FD7+SOL3, and FD9+SOL3 groups after 30 days of the trial (*p* < 0.05 or *p* < 0.01). The increase in PI for the groups of fish treated with water-soluble bacteria (SOL), namely, SOL3, SOL4 and SOL5, appeared to produce the highest value of the PI in this experiment (Figure 2M).

#### 3.4.2. mRNA Expression of Immune-Related Genes

**Figure 3:**
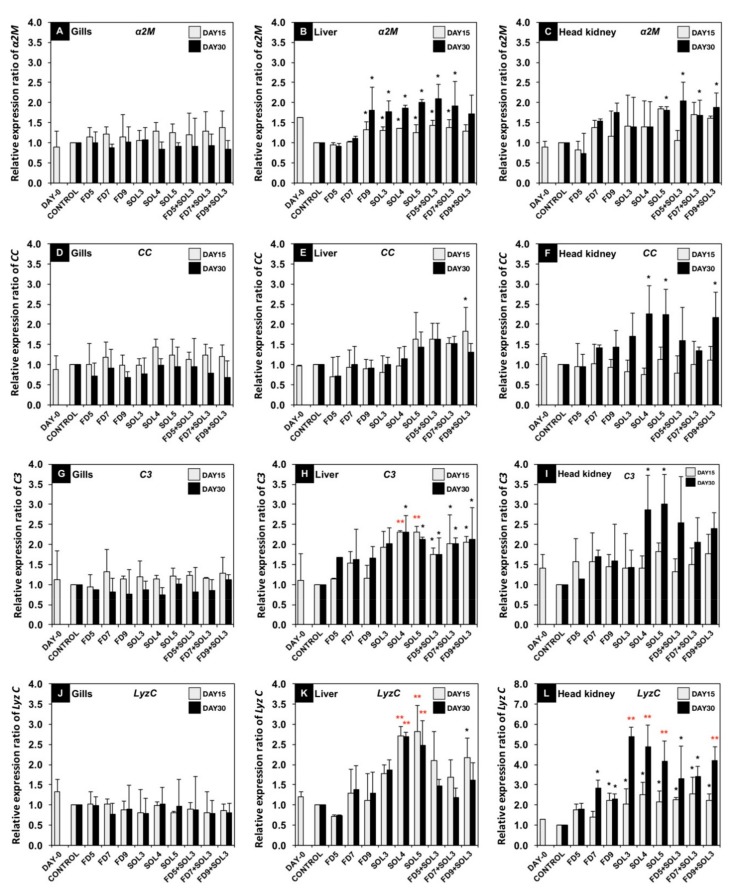

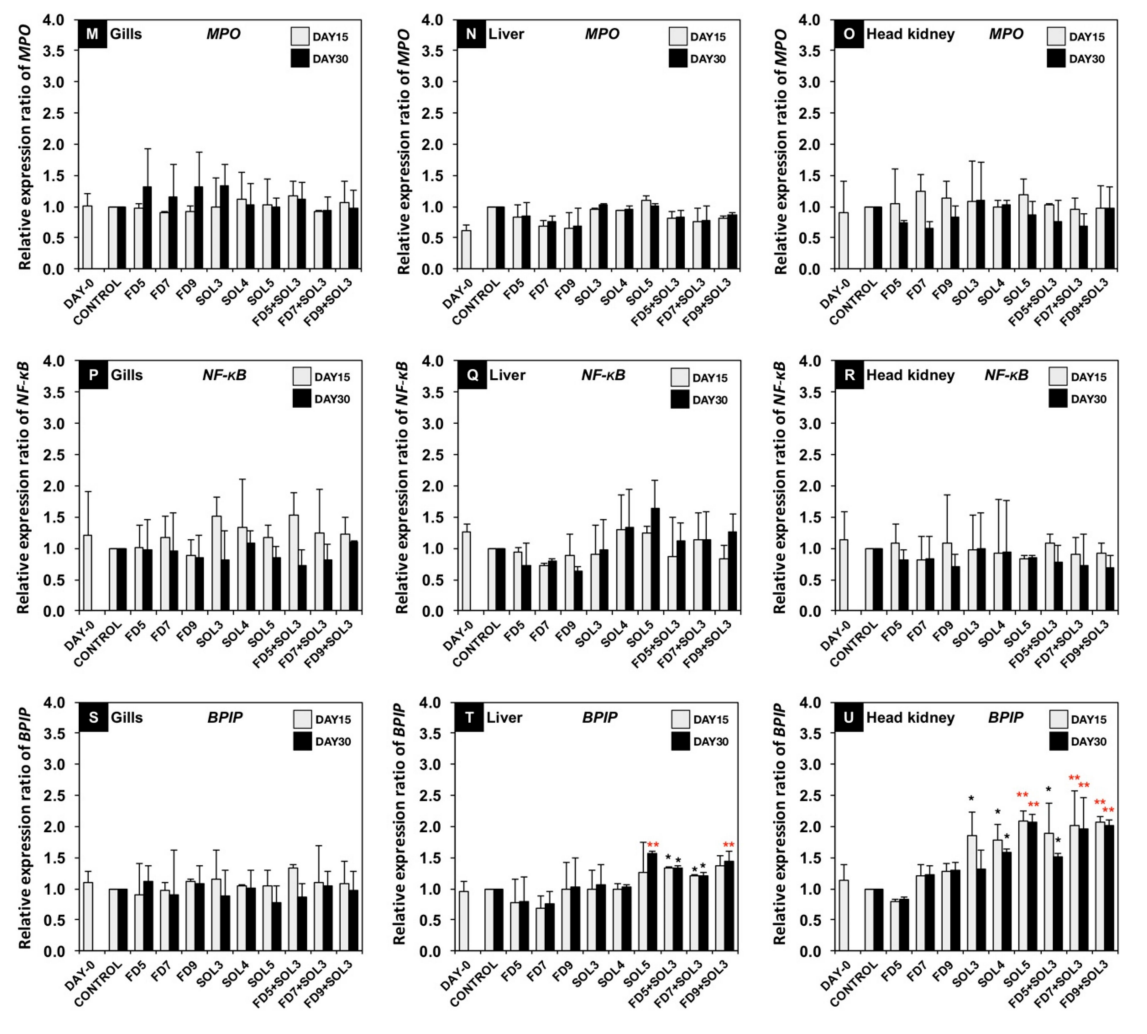
mRNA expression levels of immune-related genes, including *A2M* (**A**–**C**), *CC* (**D**–**F**), *C3* (**G**–**I**), *LyzC* (**J**–**L**), *MPO* (**M**–**O**), *NF-kB* (**P**–**R**) and *BPIP* (**S**–**U**), in the gills (A, D, G, J, M, P, S), liver (B, E, H, K, N, Q, T) and head kidney (C, F, I, L, O, R, U) of bighead catfish. The levels of statistical significance between the control and treatment groups are indicated by * (*p* < 0.05) or ** (*p* < 0.01) (*n* = 9).

The relative gene expression levels of the immune-related genes *α2M, CC*, *C3, LyzC, MPO, NF-kB* and *BPIP* in the gills, liver and head kidneys at 15 and 30 days of the trial are shown in Figure 3.

*α2M* gene expression in the liver was significantly upregulated in the treatment groups FD9, SOL3, SOL4, SOL5, FD5+SOL3 and FD7+SOL3 at both 15 and 30 days of the trial (*p* < 0.05). However, only the SOL5, FD5+SOL3, FD7+SOL3, and FD9+SOL3 groups exhibited significant upregulation of the *α2M* gene in the head kidney after 30 days of the trial compared to the control groups (*p* < 0.05). No significant differences in *α2M* gene expression in gills were detected among all groups after 15 and 30 days of the trial (Figure 3A–C).

*CC* gene expression in the FD9+SOL3 group at 15 days of the trial and in the SOL4, SOL5, and FD9+SOL3 groups at 30 days of the trial exhibited a significant increase in the liver and head kidney, respectively, compared to that in the control groups (*p* < 0.05). However, there were no significant differences in *CC* gene expression in gills among all treatment groups at 15 and 30 days of the trial (Figure 3D–F). Expression of the *C3* gene in the liver was significantly upregulated in the SOL4 SOL5, FD5+SOL3, FD7+SOL3 and FD9+SOL3 groups at 15 and 30 days of the trial compared to that in the control groups (*p* < 0.05 or *p* < 0.01). The highest significant upregulation of expression of the *C3* gene was found in the head kidneys of the SOL4 and SOL5 groups at 30 days of the trial compared to that in the head kidneys of the control groups (*p* < 0.05). No significant differences in the expression of the *C3* gene in gills were detected among all groups at 15 and 30 days of the trial (Figure 3G–I).

The highest upregulation of expression level of the *LyzC* gene was observed in the livers of the SOL4 and SOL5 groups at 15 and 30 days of the trial compared to that in the livers of the control groups (*p* < 0.05 or *p* < 0.01). Additionally, the levels were significantly increased in the head kidneys of the FD7, FD9, SOL3, SOL4, SOL5, FD5+SOL3, FD7+SOL3 and FD9+SOL3 groups at 15 days of the trial compared to those in the head kidneys of the control groups (*p* < 0.05 or *p* < 0.01). Furthermore, there were also significant increases in the FD9, SOL3, SOL4, SOL5, FD5+SOL3, FD7+SOL3 and FD9+SOL3 groups at 30 days of the trial compared to the control groups (*p* < 0.05). There were no significant differences in *LyzC* gene expression in gills among all treatment groups at 15 and 30 days of the trial (Figure 3J–L).

There were no significant differences in *MPO* and *NF-kB* gene expression in the gills, liver, or head kidney among all groups compared to the control groups at 15 and 30 days of the trial (*p* > 0.05) (Figure 3M–R). However, the expression of the *BPIP* gene was significantly upregulated in the livers of the SOL5, FD5+SOL3, FD7+SOL3 and FD9+SOL3 groups and in the head kidneys of the SOL4, SOL5, FD5+SOL3, FD7+SOL3 and FD9+SOL3 groups (*p* < 0.05 or *p* < 0.01). No significant differences in *BPIP* gene expression in the gills were observed among all groups at 15 and 30 days of the trial compared to that in the gills of the control groups (*p* > 0.05) (Figure 3S–U).

#### 3.4.3. Quantification of *Acinetobacter* KU011TH in Water, Skin, Gills and Intestine

**Figure 4:**
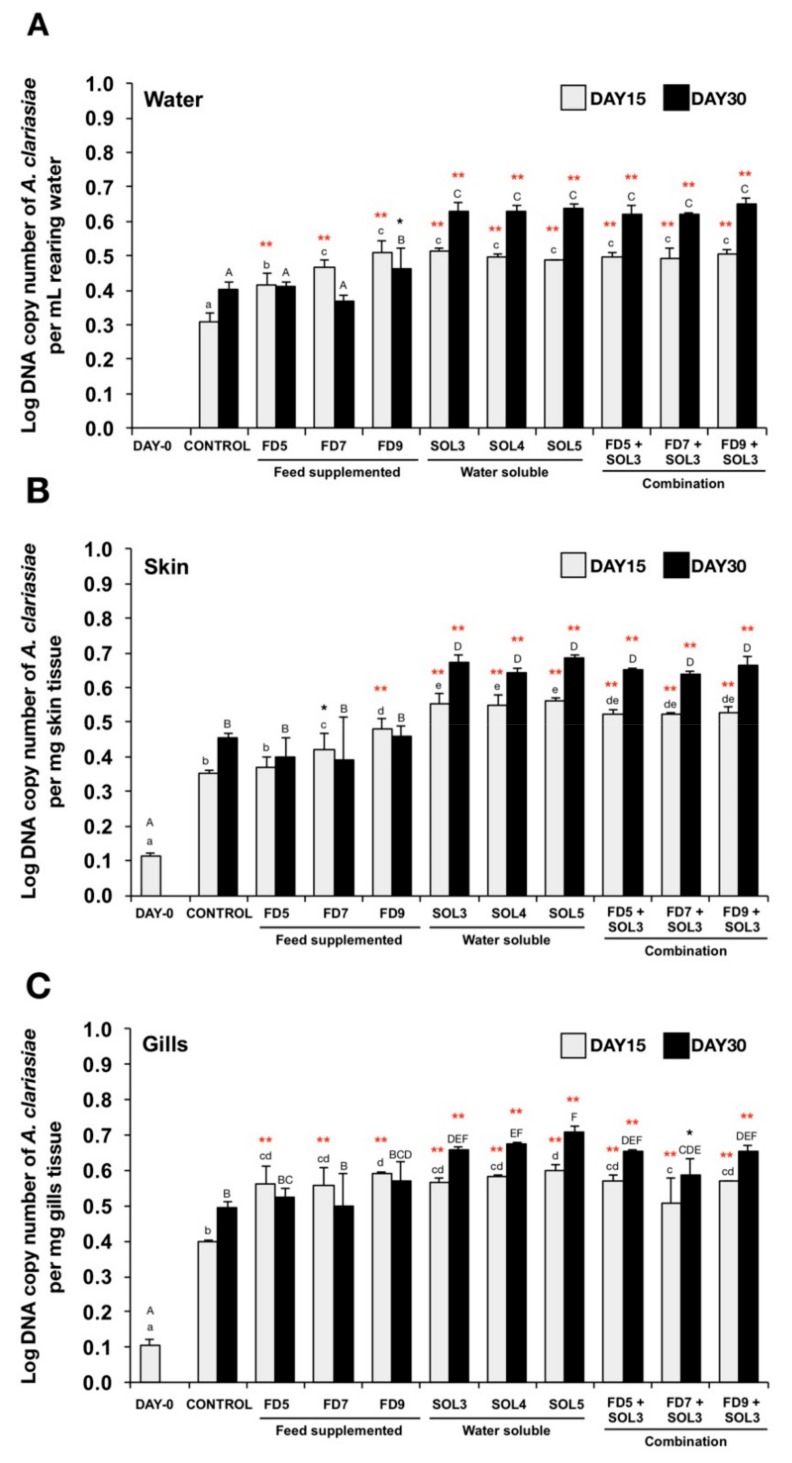
Determination of bacterial colonization using qPCR analysis of *Acinetobacter* KU011TH in rearing water (**A**), skin (**B**) and gills (**C**) of bighead catfish. Data are presented as the mean ± SEM of log DNA copy number/mg tissue or mL water (*n* = 9). Lowercase and uppercase letters indicate differences among treatment groups at 15 and 30 days of the trial, respectively. The levels of statistical significance between the control and treatment groups are indicated by * (*p* < 0.05) or ** (*p* < 0.01).

Bacterial colonization by *Acinetobacter* KU011TH in water, skin and gills (Figure 4A–C, respectively) was significantly higher in all the treatment groups at 15 days (except FD5 group) of the trial compared to that in the control groups (*p* < 0.05 or *p* < 0.01). After 30 days of the trial, the significant increase of bacterial colonization was observed in FD9, SOL3, SOL4, SOL5, FD5+SOL3, FD7+SOL3 and FD9+SOL3 groups for water. In addition, the significant increase of bacterial colonization was also observed in SOL3, SOL4, SOL5, FD5+SOL3, FD7+SOL3, and FD9+SOL3 groups for the skin and gills.

No bacterial DNA copies of *Acinetobacter* KU011TH were detected in the intestine of any experimental group.

#### 3.4.4. Skin Epithelial and Mucus Cells

**Figure 5:**
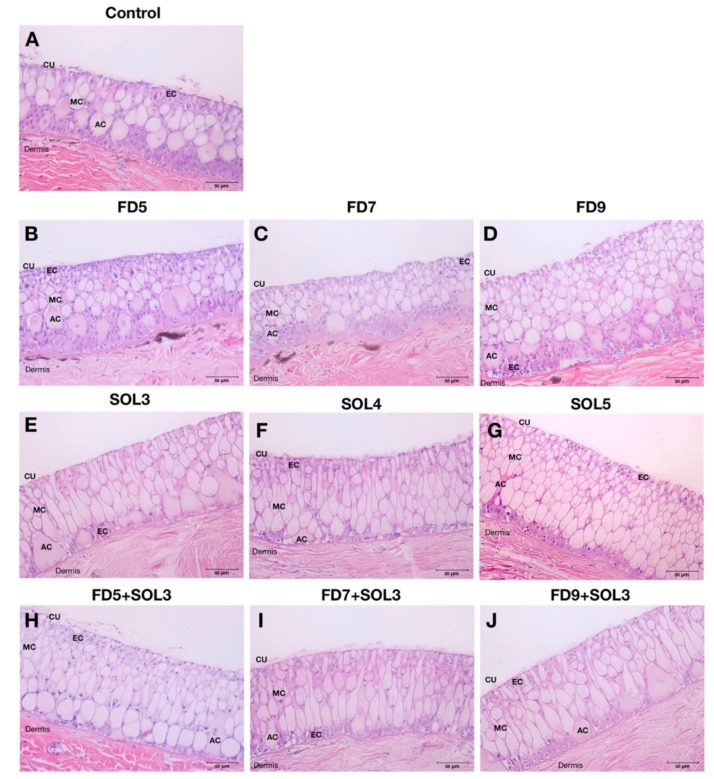
Skin histology of bighead catfish after administration of the *Acinetobacter* KU011TH bacterium for 30 days through feed (**B**–**D**), water (**E**–**G**) or a combination (**H**–**J**) compared to that of the control group (**A**). cu, skin cuticle; ec, epidermal cells; mc, mucus cells; ac, alarm cells.

**Figure 6:**
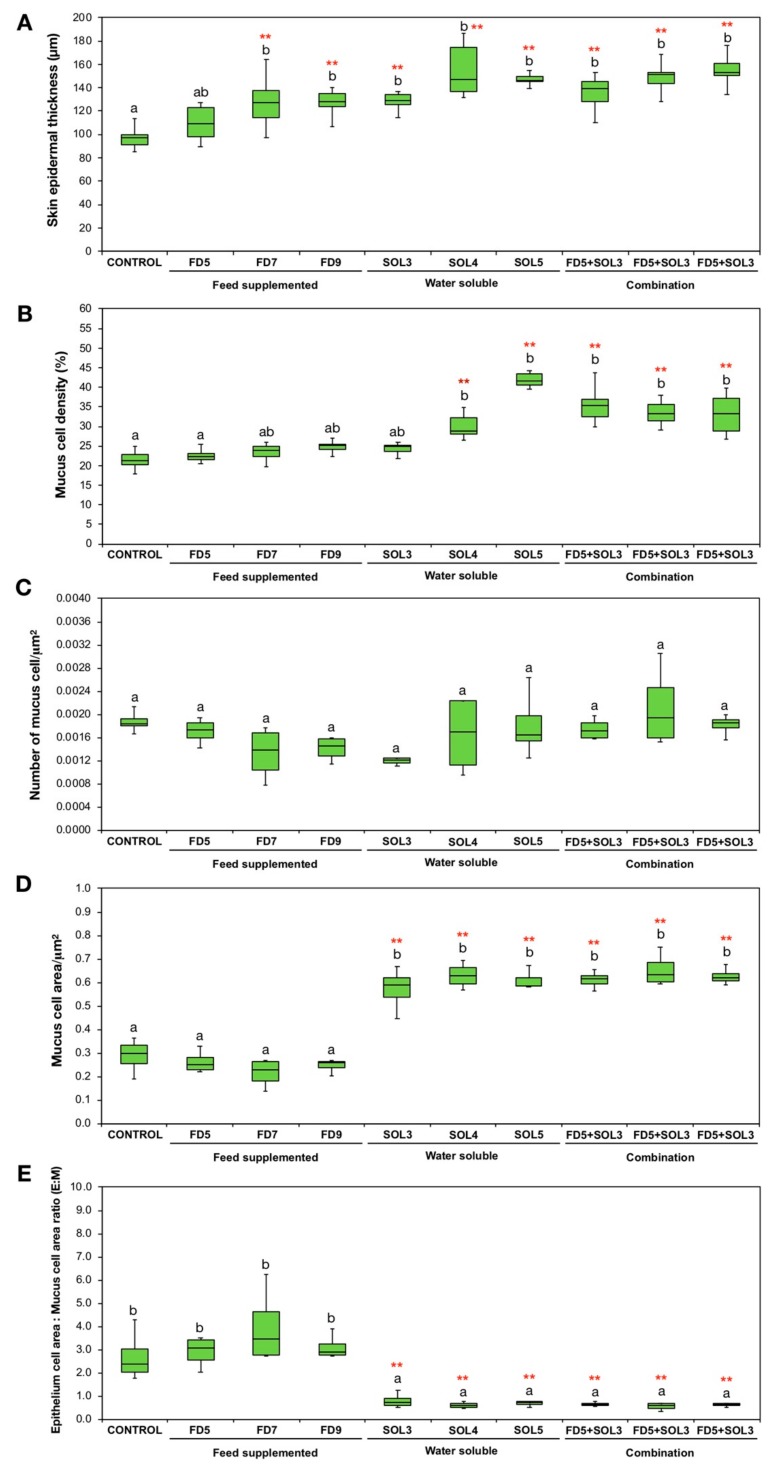
Skin physical characteristics exhibit by skin epithermal thickness (μm) (**A**), number of mucous cells/μm^2^ epithelial area (**B**), mucus cell areas/μm2 epithelial area (**C**), mucus cell density in the epithelium (%) (**D**), and the ratio of epithelial cell area to mucus cell area (E:M) (**E**) of bighead catfish (*n* = 9) after administration of the *Acinetobacter* KU011TH bacterium for 30 days. Lowercase and uppercase letters indicate differences among treatment groups at 15 and 30 days of the trial, respectively. The levels of statistical significance between the control and treatment groups are indicated as ** (*p* < 0.01).

The effects of *Acinetobacter* KU011TH on skin mucus cells of *C. macrocephalus* are presented in Figure 5 and Figure 6. After 30 days of the trial period, there was a significant increase in epidermal skin thickness in the experimental groups FD7, SOL3, SOL4, SOL5, FD5+SOL3, FD7+SOL3, and FD9+SOL3 compared to the control groups (*p* < 0.01), but no significant difference was observed between the FD5 and control groups (*p* > 0.05) (Figure 6A). The increases in mucus cell density in the skin epithelium were highly significant in the SOL4, SOL5, FD5+SOL3, FD7+SOL3, and FD9+SOL3 groups compared to those in the control groups (*p* < 0.01) (Figure 6B). No significant differences in the number of mucus cells per square micrometer of skin epithelium were detected among all the treatment groups (*p* > 0.05) (Figure 6C). Nevertheless, the mucus cell area per square micrometer of skin epithelium and E:M ratio showed highly significant differences in the SOL3, SOL4, SOL5, FD5+SOL3, FD7+SOL3, and FD9+SOL3 groups compared to those in the control groups (*p* < 0.01). No significant differences were observed among the control, FD5, FD7, and FD9 groups (*p* > 0.05) (Figure 6D–E).

#### 3.4.5. Histopathology of the Skin, Gills, Intestine and Liver

**Figure 7:**
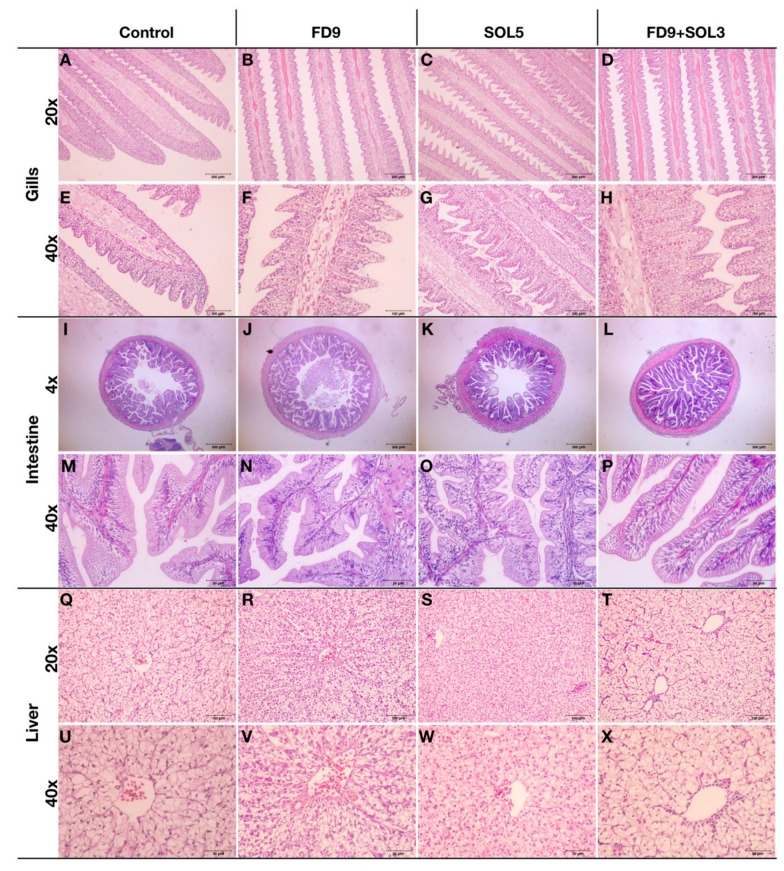
Histological sections of the gills (**A**–**H**), intestine (**I**–**P**) and liver (**Q**–**X**) of bighead catfish after administration of an *Acinetobacter* KU011TH supplemented diet with 1 × 10^9^ CFU/kg diet (FD9) (B, F, J, N, R, V), water-soluble bacteria at 1 × 10^5^ CFU/mL rearing water (SOL5) (C, G, K, O, S, W), or a combination of the *Acinetobacter* KU011TH supplemented diet at 1 × 10^9^ CFU/kg diet and water-soluble bacteria at 1 × 10^3^ CFU/mL rearing water (FD9+SOL3) (D, H, L, P, T, X) for 30 days compared to those of a control group (A, E, I, M, Q, U). The histological sections illustrate only the highest concentration of administered probiotics in each experimental treatment group since there were no significant differences among the other groups.

The effects of *Acinetobacter* KU011TH on histopathological changes in the skin, gills, head kidney and liver are presented in Figure 5 and Figure 7. After 30 days of the trial, no histological changes in skin, gills, intestine, and liver tissues were observed upon examination, which revealed neither clinical signs nor pathological lesions in all treatment groups compared to the control groups. Notably, the intestinal sections of all catfish in the FD9+SOL3 group showed enlargement of the intestinal mucosal epithelium through organization of villi mucosal folds compared to those of the treatment groups.

#### 3.4.6. Resistance against *A. hydrophila*

**Figure 8:**
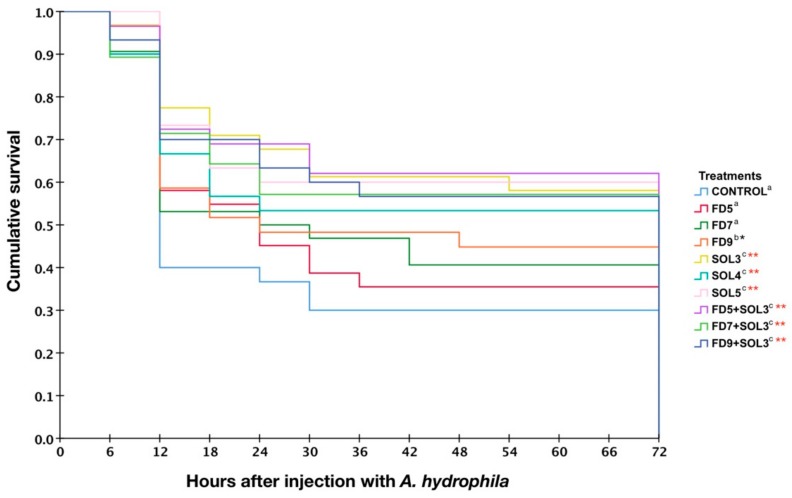
Survival analysis of bighead catfish administered *Acinetobacter* KU011TH for 30 days after challenge with *A. hydrophila*. Data and survival plots were examined using the Kaplan-Meier method. Letter superscripts indicate that the level of statistical significance between the control group and treatment group is *p* < 0.05. The levels of statistical significance between the control and treatment groups are indicated as * (*p* < 0.05) or ** (*p* < 0.01) (*n* = 30).

The survival rates of *C. macrocephalus* after challenge with *A. hydrophila* are shown in Figure 8. After injection, rapid mortality was observed from h 12 to 30, stabilizing at hr 54 until the end of observation at day 15. The disease resistance of fish administered *Acinetobacter* KU011TH against *A. hydrophila* demonstrated that the survival of fish in groups FD9, SOL3, SOL4, SOL5, FD5+SOL3, FD7+SOL3, and FD9+SOL3 was significantly higher than that in the control, FD5, and FD7 groups (*p* < 0.05 or *p* < 0.01). The survival rates of the FD9, SOL3, SOL4, SOL5, FD5+SOL3, FD7+SOL3 and FD9+SOL3 groups were 48.33 ± 2.36%, 56.70 ± 0.63%, 53.13 ± 4.42%, 58.81 ± 7.74%, 57.69 ± 5.44%, 57.14 ± 10.10%, and 56.67 ± 4.71%, respectively. Meanwhile, the survival rates of the control, FD5, and FD7 groups were 30.00 ± 4.71%, 25.63 ± 7.95%, and 34.76 ± 11.45%, respectively.

## 4. Discussion

The genus *Acinetobacter* is currently comprised of fifty-five distinct species that have been originally isolated from various sources, such as human clinical specimens, invertebrate and vertebrate animals, vegetables, soil and water [34,35]. Although research on the genus *Acinetobacter* has been particularly focused on some species that have effects on human health, especially *A. baumannii,* the beneficial effects of most of the remaining species on their hosts have never been addressed to date. The present study provides strong evidence of a novel probiotic candidate of the *Acinetobacter* genus, *Acinetobacter* KU011TH, which has been proven to be safe and has numerous beneficial effects on its host, *C. macrocephalus*. Administration of *Acinetobacter* KU011TH to *C. macrocephalus* led to a significant increase in growth performance, innate immune responses, skin physical immunity, immune-related gene expression, and disease resistance against the *A. hydrophila* pathogen. This study is the first report of the application of the genus *Acinetobacter* as a potential probiotic in vertebrates.

The importance of toxicity and pathogenicity assessments of applied probiotics has been recognized, and these assessments are required for applications in hosts or living organisms. The safety assessment of *Acinetobacter* KU011TH probiotics in the scaleless host catfish has scientifically been performed thoroughly by both LD_50_ and LC_50_ determination. No mortality, clinical or pathological features of swimming behavior and changes in skin and gill appearances were observed during the trials. Thus far, the strain is considered “generally recognized as safe” (GRAS) according to the United States Food and Drug Administration (FDA) designation [36]. The results suggest that the strain is safe for the host and can be applied as a potential probiotic for further study.

The application of probiotics in the aquaculture industry has been successfully achieved in various aquatic animals, especially fish and shellfish [10,16,23]. Moreover, various factors associated with probiotic administration, such as probiotic sources, administration methods, doses, and durations, have been established. Several microorganisms that are normally found in the digestive tracts, skin, gonads and culture systems, such as rearing water and sediment, are often used as probiotics in the aquaculture industry [10,16,37]. Potential probiotics could be administered via the feed as feed additives, directly into the rearing water or as a combination of feed supplementation and administration via rearing water [10,26,38]. Furthermore, a wide range of doses has been commonly applied, such as 1 × 10^5^ to 1 × 10^9^ CFU/g feed and 1 × 10^5^ to 1 × 10^9^ CFU/mL water for feed and rearing water additives, respectively [26,37,39,40]. Probiotics could be practically administered over a wide range of time periods, from two weeks up to eight months [10,16,23,37,41,42]. Unfortunately, there is no evidence to prove which application methods are the best strategies for aquatic animals. Therefore, understanding the nature of probiotic strains and host specificity along with the modes of action may lead to optimization of the appropriate administration methods that can help provide favorable benefits to the hosts. 

The viable shelf life of *Acinetobacter* KU011TH stored at 4 °C was stable during weeks 1–4 but seemingly decreased at week five and stabilized during weeks 7–10. At the end of the experiment, the bacterial concentrations were recorded and were seen to be clearly decreased by approximately 50%. However, the efficacy of *Acinetobacter* KU011TH in the control of *A. hydrophilia* remained strong compared to that of any previous storage time. This result suggested that important components used to inhibit the target pathogen were functionally maintained in all remaining bacterial cells, even though half of them were sacrificed. Therefore, further study and validation of powerful preservation methods are required to prolong shelf life for full-scale industrial application. 

Growth performance enhancement is the most promising beneficial effect of probiotic administration on the host because it may lead to enhanced appetite and increased growth. According to our findings, a long-term 120-day administration of *Acinetobacter* KU011TH via a combination of feed and water at concentrations of 1 × 10^9^ CFU/kg diet and 1 × 10^3^ CFU/mL, respectively, led to significantly increased WG, ADG, RGR and SGR and decreased FCR for body weight compared to those of the control groups (*p* < 0.05). These improvements could be attributed to the production of exogenous enzymes involved in feed utilization by the strain. In addition, perhaps the strain produces some essential vitamins for growth promotion or may produce enzymes involved in feed utilization [26,43]. Similar to our study, administration of several probiotic bacteria has been proven to promote growth in several fish species, including channel catfish (*Ictalurus punctatus*), striped catfish (*Pangasianodon hypophthalmus*), pla-mong (*Pangasius bocourti*), grouper (*Epinephelus coioides),* rainbow trout (*Oncorhynchus mykiss*), and starry flounder (*Platichthys stellatus*) [24,25,26,27,44,45,46,47].

In the long-term experimental trial, based on the obtained information, application of *Acinetobacter* KU011TH as probiotics for catfish rearing was obviously found to increase the catfish growth rate solely upon administration via a combination of water and diet supplementation (FD9 + SOL3). However, colonization by *Acinetobacter* KU011TH in this study was not detected in the intestines of the treated groups or the control group by qPCR. The results suggest that the probiotic bacterium cannot survive and colonize during passage through the GI tract. Indeed, this bacterium can be destroyed by the acidity, digestive enzymes and lack of oxygen in the GI tract, which is not the original source of this bacterium. Nevertheless, the results also suggest that the strain may influence GI tract performance by sacrificing itself and providing some important nutrients for the host and its microbiota. Their interactions could extensively promote GI mucosal cell proliferation to give rise to all cell lineages, such as epithelial or goblet cells, and enhance mucosal barrier function, as found in a previous study [48,49,50] and in this study in the FD9+SOL3 fish treatment group (Figure 7L,P). This effect must be due to unknown important factors that improve the optimal balance of the gut microbiome and nutrient utilization of both the host and microorganisms, which consequently influences and elevates host growth eventually. The results suggest that this bacterium is a beneficial normal flora microorganism found in the body and in the environment surrounding the catfish.

Modulation of the immune system is an important characteristic of probiotic bacteria that could enhance innate immune responses in both humoral and cellular defense mechanisms that act as a first line of protection against detrimental substances or pathogens [23,41]. Probiotics interact with humoral defense mechanisms by enhancing various serum enzyme activities associated with bacteriolysis against Gram-positive and Gram-negative bacteria, such as lysozyme, respiratory burst, as well as complement and bactericidal activities. In addition, probiotics could also actively stimulate innate cellular defenses through immune cells, such as phagocytic cells, including monocytes, macrophages and neutrophils, to enhance innate immune responses [23]. In our findings, the probiotic bacteria could markedly increase both innate humoral and cellular defenses through lysozyme activity, respiratory burst activity, ACH_50,_ serum bactericidal activity, PA and PI in fish administered *Acinetobacter* KU011TH through feed, water or a combination of the two routes. Moreover, the *Acinetobacter* KU011TH probiotics demonstrated upregulatory effects on the expression of the *α2M, CC, C3, LyzC* and *BPIP* genes in catfish livers and head kidneys [24].

There are associations with the regulation of nonspecific humoral factor responses, which are also regarded as biological markers for determining fish health [22,23]. In general, the most effective strategy for activating immune responses by probiotic bacterium administration in previous reports was typically found to be administration via water, a combination of feed and water, or feed [26,38,51,52]. However, most of the studied probiotics exhibited the highest efficacy via only feeding with probiotic-supplemented diets because most of these probiotics were spore-forming organisms that were originally isolated from the digestive tracts of fish. In contrast, the *Acinetobacter* KU011TH probiotic bacterium was originally isolated from the skin mucus of bighead catfish and can be beneficial to the host in all practical applications in the aquaculture industry. Although the mode of action could not be elucidated in this study, the most likely mechanism can be hypothesized. Lipopolysaccharide (LPS) and peptidoglycan (PGN) are classified as abundant pathogen-associated molecular patterns (PAMPs) found on the cell surfaces of Gram-positive and Gram-negative bacteria, respectively [53]. LPS and PGN from probiotic bacteria have been widely reported as immunostimulants in several aquatic animals and successfully enhance innate humoral and cellular immune responses. The current study suggests that *Acinetobacter* KU011TH provides LPS to activate and improve innate immune responses in both humoral and cellular defenses in bighead catfish. Such probiotic water additive-induced innate immunity could effectively increase the interactions between PAMPs and nonspecific immune receptors by triggering fish mucosal immunity, such as in the gills, nares and skin, which are the primary surface organs that are under constant exposure to the environment [54]. Indeed, the interaction can also increase the interactions of the intestinal epithelium and immune cells in the GI tract via accidental ingestion of bacteria from the surrounding water, resulting in local activation of the fish innate immune system.

Recently, immunomodulatory features of probiotics in aquatic animals were suggested by many studies that be associated with mucosal immunity, which plays a key role in the first-line defense against harmful pathogens and is thus considered to be the most active immunological site in fish [25,54]. Mucosal immunity of fish is driven by mucosa-associated lymphoid tissues (MALTs). These sites are divided into gut-associated lymphoid tissues (GALTs), skin-associated lymphoid tissues (SALTs), gill-associated lymphoid tissues (GIALTs) and, recently, nasopharynx-associated lymphoid tissues (NALTs) [54]. Moreover, colonization by probiotic bacteria represents the persistence of living microbial cells that adhere to favorable surfaces, including in rearing water and mucosal surfaces, such as the gut, skin and gills. The current study also demonstrated that a probiotic bacterium, *Acinetobacter* KU011TH, could colonize rearing water and the skin, and gills but not the intestine. This finding suggested that *Acinetobacter* KU011TH is a probiotic microbe that influences MALTs, especially the SALT, of the scaleless bighead catfish. *Acinetobacter* KU011TH affects the host by maintenance of the microbiota and mucosal surface and reinforcement of immunity. The administration of rich, potent probiotics to the mucosal surface has become a prominent mode of immunomodulation and control of infectious disease in aquatic animals [25,54,55,56].

In fish, skin and skin mucus cells are directly influenced by SALTs, which are classified as a first-line defense of innate immune responses to both physical and chemical approaches. Fish skin cells are typically localized with a large number of different nonspecific immune cells [57]. However, the skin of all fish species is constructed similarly to that of other vertebrates, consisting of two basic layers, namely, the epidermis and dermis. Indeed, the mucus cells, club cells, alarm cells and sensory cells are abundantly embedded in the epidermal layer [57]. Recently, mucus cells have been reported as the major immunocompetent cells that contain a rich assortment of biologically active components, especially antimicrobial proteins (AMPs), such as alkaline phosphatase, cathepsin B, complement, transferrin, lysozyme, hepcidin, cathelicidins, apolipoproteins, piscidin, pleurocidin and C-reactive protein [58,59]. During the last decade, it has been reported that several types of AMPs, including H2A, H1, H6, cathelicidins and piscidin, are produced by skin mucus cells of rainbow trout (*Oncorhynchus mykiss*) [55,56]. Additionally, the skin mucus of *Clarias batrachus* exhibited strong inhibitory activity against five pathogenic bacteria, namely *Escherichia coli*, *A. hydrophila*, *Pseudomonas aeruginosa*, *Vibrio fischeri* and *Vibrio anguillarum*, on agar plates [60]. Several recent studies have demonstrated that enhancement of SALT performance leads to increased skin mucus levels, skin AMP levels, skin epidermal thickness, number of mucus cells and mucus cell areas in fish fed a probiotic-supplemented diet, such as angelfish (*Pterophyllum scalare*), common carp (*Cyprinus carpio*), European sea bass (*Dicentrarchus labrax*) and Atlantic salmon (*Salmo salar* L.) [30,40,61,62]. Similar to the results of our study, significantly increased epidermal skin thickness, mucus cell density, and mucus cell areas and decreased E:M ratios were observed in fish administered *Acinetobacter* KU011TH through feeding, as a water additive and their combination for 30 days. It has been suggested that the probiotic *Acinetobacter* KU011TH may exert an influence on the SALT, GALT, NALT and GIALT performances of bighead catfish, but the mechanisms involved remain unknown.

Notably, histological analyses of the skin and intestine strongly confirmed the histoarchitectural performance of SALTs in all treatment groups and GIALTs in the FD9+SOL3 group compared to those in the control group. Furthermore, probiotic *Acinetobacter* KU011TH administration was associated with enhanced innate immune responses and increased skin physical immunity without negative effects on the histoarchitectures of the target tissue organs, including the skin, gills, intestine and liver, of fish administered the probiotic for 30 days.

The final proof of the magnitude of the effect of the probiotic bacteria on aquatic animals was performed by health assessments through challenge tests with specific harmful pathogens that cause infectious diseases. Fish typically respond to any pathogens via the entire innate and adaptive immune responses with humoral and cellular mechanisms to contain infectious diseases [63]. The initial responses are primarily driven by nonspecific humoral factors, including growth-inhibiting substances, antimicrobial peptides, complement peptides, and nonspecific cellular responses, such as phagocytosis. Furthermore, in certain infectious disease outbreaks, strong potential responses [37] associated with adaptive immune responses are required, which are driven by mainly immunoglobulins [64]. In accordance with our finding, an increased survival rate after challenge with *A. hydrophila* was observed in fish administered *Acinetobacter* KU011TH in all experimental treatment groups. Our findings suggest that administration of *Acinetobacter* KU011TH by feeding, as water additive and their combination activates fish immunity through several mechanisms of both innate and adaptive immune responses, leading to disease resistance against *A. hydrophila*. Furthermore, previous studies of probiotic administration in fish have shown successfully increased survival rates after challenge with pathogens in several fish, including *O. niloticus, O. mykiss* and *Labeo rohita.* These administered probiotics were also found to enhance innate immune responses, including respiratory burst, lysozyme and phagocytic activities [39,43,65].

Among application routes, water supplementation with 1 × 10^3^–10^5^ CFU/mL *Acinetobacter* KU011TH was the most predominant method that strongly elevated both innate immune parameters and immune-gene e × pression. Feed supplementation with 1 × 10^5^−10^7^ CFU/mL probiotic was less effective than the other routes but exhibited significant differences compared to the control group. Surprisingly, the combination of the water- (1 × 10^3^ CFU/mL) and feed-supplemented (1 × 10^5^–10^9^ CFU/kg diet) routes did not have a synergistic effect over the others, but the effect remained stronger than that of the control and feed-supplemented groups. When considered together with pathogen challenge, the data clearly showed that the combination of water supplemented with 10^3^ CFU/mL and feed supplemented with1 × 10^3^, 1 × 10^5^ and1 × 10^7^ CFU/kg diet effectively enhanced catfish resistance against the harmful pathogen. These results suggested that water-soluble application of the viable probiotic at concentrations ranging from 1 × 10^3^–10^5^ CFU/mL at three-day intervals for 30 days is sufficient to elevate the catfish immune system to safeguard the fish from *A. hydrophila* infection.

Additionally, supplementation with *Acinetobacter* KU011TH via both rearing water and feed in the short trial clearly demonstrated that all skin characteristic parameters were significantly improved compared to those of the control and feed-supplemented probiotic treatment groups. This result indicated that adding this bacterium to fish feed did not lead to skin alteration, and no synergistic effect of the combinatorial application of this bacterium was observed. The improvement of skin characteristics may also greatly induce first-line defense mechanisms to effectively protect catfish from several contaminating pathogens in water. Furthermore, the impenetrability of mucosal immunity at the gills, nares, and skin of the scaleless fish, which is crucial for preventing invasion by all pathogens from the external environment, may be properly strengthened after probiotic application. The use of this probiotic will be a crucial and novel application for disease prophylaxis strategies during walking catfish aquaculture.

## 5. Conclusions

The importance of the achievements from the use of probiotics in catfish cultures has been established in this study. *Acinetobacter* KU011TH is a novel probiotic bacterial species in the genus *Acinetobacter* that was originally isolated from the skin mucus of bighead catfish. From previous research, this strain exhibited high efficacy against several pathogenic bacteria of freshwater fish *in vitro,* such as *A. hydrophila, F. columnare, F. roseus, S. agalactiae, S. warneri* and *E.* *tarda* [12]. To date, *Acinetobacter* KU011TH has appreciably enhanced catfish health performance, increasing growth parameters, innate humoral and cellular responses, immune-related gene expression, skin physical defense and disease resistance. Additionally, *Acinetobacter* KU011TH has been proven to be a nonpathogenic probiotic toward *C. macrocephalus* and does not negatively affect any histopathological changes in the target organs. *Acinetobacter* KU011TH in the catfish aquaculture industry could be practically applied as an additive in rearing water or as a combination of water additive and feed additive for enhanced benefits. Certainly, among the probiotic candidates for aquatic animals suggested during this decade, *Acinetobacter* KU011TH could be a noteworthy species with applications in the catfish industry, achieving a reduction in disease outbreaks and antibiotic application, which have been longstanding issues for the aquaculture industry worldwide.

## Figures and Tables

**Table 1:** Primers used for quantification of *Acinetobacter* KU011TH colonization and immune-related gene expression by qPCR and qRT-PCR of bighead catfish.

Gene	Primer Names	Nucleotide Sequences (5’-3’)	Amplicon Size (bp)	T_m_ (°C)	Accession Number
*Acinetobacter* KU011TH *gyrB*	*Ac_gyrB_F* *Ac_gyrB_R*	GGCGTGCGTATTGTTTTACGTGAT CAATACCGTTTTCTGTATCTGCGG	154	59	MG950236
Alpha-2-macroglobulin (*A2M*)	*Cm*_*A2M*_F *Cm*_*A2M*_R	TACTTCTCTACAATGCCCCTACAC GATCGGCTATGAACCCTGATAAGA	192	60	SRS1284019
CC chemokine (*CC*)	*Cm*_*CC*_F *Cm*_*CC*_R	TCTTAACAATGAAGCCTTGCTGTG ACATGAAGATGAACCGTGTGTTTT	196	59	
C3 complement (*C3*)	*Cm*_*C3*_F *Cm*_*C3*_R	GAGCAAATACTTTGGCAACATACG GTGAGGCTATCCAACACATTCAGA	135	60	
Lysozyme C (*LyzC*)	*Cm*_*LYZc*_F *Cm*_*LYZc*_R	TGCTAAACAGTATGATCGGTGTGA TATCTGGAAAATGCCGTAGTCTGT	178	60	
Myeloperoxide (*MPO*)	*Cm*_*MPO*_F *Cm*_*MPO*_ R	ACTTCCTCAACTGCAGAAGTATCC GAGGCTTCTCATTACTGGCTGAT	124	60	
Nuclear factor kappa B (*NF-κB*)	*Cm*_*NFKb*_F *Cm*_*NFKb*_R	CGTACCCTCAGGTTAAGATCTGTC TACCAAGGTTAGGGAAACTGATGG	183	60	
Bactericidal/permeability-increasing protein (*BPIP*)	*Cm*_*BPIP*_F *Cm*_*BPIP*_R	TCGACTGAACACAAAGACATTTGG GCTGAATAGCGTAAGCAGTAATGG	170	59	
Beta-actin *(β-actin)*	*Cm*_ *βActin*_F *Cm*_ *βActin* _R	GTCCGTGACATCAAGGAGAAGCTC GGACTCCATACCCAGGAAAGATGG	189	59	

**Table 2:** Bacterial shelf life analyses and efficacy of the probiotic *Acinetobacter* KU011TH activity against the pathogen *A. hydrophila* after storage at 4 °C for ten weeks.

Week	Total Viable Count ( × 10^7^ CFU/mL)	Percentage Change *	Clearance Zone Diameter (cm)
0 (control)	6.77 ± 0.28 ^d^	0	1.76 ± 0.03 ^a^
1	6.49 ± 0.42 ^d^	−4.19%	1.73 ± 0.03 ^a^
2	6.56 ± 0.38 ^d^	−3.15%	1.69 ± 0.09 ^a^
3	6.44 ± 0.23 ^d^	−4.87%	1.66 ± 0.03 ^a^
4	6.46 ± 0.42 ^d^	- 4.53%	1.66 ± 0.05 ^a^
5	5.34 ± 0.22 ^c^	−21.17%	1.68 ± 0.04 ^a^
6	4.45 ± 0.29 ^b^	−34.22%	1.74 ± 0.10 ^a^
7	3.68 ± 0.50 ^a^	−45.69%	1.67 ± 0.06 ^a^
8	3.43 ± 0.03 ^a^	−49.29%	1.66 ± 0.03 ^a^
9	3.64 ± 0.22 ^a^	−46.28%	1.72 ± 0.05 ^a^
10	3.21 ± 0.04 ^a^	−52.63%	1.70 ± 0.04 ^a^

Data are expressed as the mean ± SEM. Letters in the same column indicate that the level of statistical significance between week 0 (control) samples and the tested samples in different time intervals is *p* < 0.05. *, percentage changes in the viable bacterial count in each week were calculated compared to those in week 0 (control).

**Table 3:** Of various administration routes of *Acinetobacter* KU011TH on the growth performance of bighead catfish at 120 days (*n* = 30).

Growth Performance Parameters	Experimental Groups									
	Control	Probiotic-Supplemented Diet (FD)			Water-Soluble Probiotics (SOL)			Combination of FD and SOL		
		FD5	FD7	FD9	SOL3	SOL4	SOL5	FD5 + SOL3	FD7 + SOL3	FD9 + SOL3
**1. Body weight analysis**										
1.1 AGR: WG (g /120 days)	19.45 ± 0.57 ^a^	23.71 ± 1.85 ^a^	24.48 ± 1.24 ^a^	23.57 ± 5.48 ^a^	21.99 ± 4.13 ^a^	21.51 ± 4.06 ^a^	23.16 ± 4.17 ^a^	24.34 ± 1.26 ^a^	22.16 ± 0.81 ^a^	25.19 ± 0.19 ^b^
1.2 AGR: ADG (g/day)	0.16 ± 0.00 ^a^	0.20 ± 0.02 ^a^	0.20 ± 0.01 ^a^	0.20 ± 0.05 ^a^	0.18 ± 0.03 ^a^	0.18 ± 0.03 ^a^	0.19 ± 0.03 ^a^	0.20 ± 0.01 ^a^	0.18 ± 0.01 ^a^	0.24 ± 0.00 ^b^
1.3 RGR (% in 120 days)	177.37 ± 2.25 ^a^	205.23 ± 14.48 ^ab^	224.61 ± 0.56 ^ab^	209.72 ± 36.41 ^ab^	207.46 ± 45.14 ^ab^	195.17 ± 32.34 ^ab^	210.55 ± 35.08 ^ab^	219.20 ± 0.66 ^ab^	202.55 ± 12.83 ^ab^	240.53 ± 7.06 ^b^
1.4 SGR (%/day)	0.369 ± 0.003 ^a^	0.404 ± 0.017 ^ab^	0.426 ± 0.001 ^ab^	0.408 ± 0.043 ^ab^	0.405 ± 0.053 ^ab^	0.391 ± 0.040 ^ab^	0.409 ± 0.041 ^ab^	0.420 ± 0.001 ^ab^	0.400 ± 0.015 ^ab^	0.443 ± 0.008 ^b^
1.5 FCR	3.09 ± 0.09 ^a^	2.74 ± 0.20 ^a^	2.75 ± 0.12 ^a^	2.62 ± 0.6 ^a^	2.78 ± 0.52 ^a^	2.84 ± 0.54 ^a^	2.63 ± 0.47 ^a^	2.77 ± 0.13 ^a^	2.71 ± 0.10 ^a^	2.38 ± 0.02 ^b^
**2. Body length analysis**										
2.1 AGR: TLG (cm/120 days)	12.65 ± 0.39 ^a^	10.78 ± 0.68 ^a^	10.09 ± 1.71 ^a^	12.57 ± 2.17 ^a^	11.13 ± 0.03 ^a^	10.82 ± 0.45 ^a^	14.16 ± 2.52 ^a^	12.55 ± 0.48 ^a^	11.71 ± 0.28 ^a^	12.00 ± 0.18 ^a^
2.2 AGR: ADG (cm/day)	0.11 ± 0.00 ^a^	0.09 ± 0.01 ^a^	0.08 ± 0.01 ^a^	0.10 ± 0.02 ^a^	0.09 ± 0.00 ^a^	0.09 ± 0.00 ^a^	0.12 ± 0.02 ^a^	0.10 ± 0.00 ^a^	0.10 ± 0.00 ^a^	0.10 ± 0.00 ^a^
2.3 RGR (% in 120 days)	169.04 ± 2.16 ^a^	159.21 ± 15.38 ^a^	160.03 ± 15.93 ^a^	163.78 ± 42.03 ^a^	1520.94 ± 5.61 ^a^	139.46 ± 8.98 ^a^	168.04 ± 37.60 ^a^	147.89 ± 16.04 ^a^	151.82 ± 9.92 ^a^	155.94 ± 2.05 ^a^
2.4 SGR (%/day)	0.36 ± 0.00 ^a^	0.28 ± 0.03 ^a^	0.28 ± 0.03 ^a^	0.35 ± 0.06 ^a^	0.32 ± 0.01 ^a^	0.32 ± 0.01 ^a^	0.38 ± 0.05 ^a^	0.33 ± 0.02 ^a^	0.33 ± 0.01 ^a^	0.34 ± 0.00 ^a^

Data are expressed as mean ± SEM. Data in the same rows with different letters indicate that the level of statistical significance between the control and treatment groups is *p* < 0.05.
